# Acute ischaemic stroke: highlighting the need for early intervention

**Published:** 2013-04

**Authors:** Girish Modi

**Affiliations:** Department of Neurosciences, School of Clinical Medicine, Faculty of Health Sciences, University of the Witwatersrand, Johannesburg, South Africa

If only the brain was like the heart where losing a ‘bit’ from ischaemia and infarction would have little effect on its function, the treatment of stroke in the acute setting would not be such a public health issue. The effects of stroke on an individual and the burden its leaves on families and society are enormous and costly.

Stroke remains the third leading cause of death worldwide. The annual death toll on stroke is reported as being approximately 7.8 million.^1^ Despite current treatment options and interventions, this number is projected to rise and reach almost eight million in the next 15 years. In developed countries the cost of stroke treatment ranges from $500–150 000.^2^ This wide range is presumably related to the greater use of mechanical interventions in some countries as opposed to predominantly pharmaceutical interventions in others. Unfortunately, in developing countries, cost estimates are not readily available. The only reported data comes from Togo where a cost of EUR400 is estimated.^3^

Definitive treatment that would completely reverse an ischaemic event to the brain remains the holy grail of neurology. In the review by Drs Jivan, Ranchod and Modi, a PUBMED search and analysis of data on the management of acute ischaemic stroke (AIS) from 1995 to 2012 is presented. The interventions described include intravenous recombinant tissue plasminogen activator (IV r-tPA)-induced thrombolysis, intraarterial (IA) thrombolysis and the controversial aspects of clotremoval treatments.

The conclusions are simple in that the gold standard for treating AIS remains IV r-tPA. The important facts in this regard are that the other intravenously administered agents, including streptokinase, reteplase, urokinase, anistreplase and staphylokinase, show no benefit and should be avoided in routine clinical practice.^4^ The data on IV r-tPA from the ECASS 3 trial indicated that for every 100 patients treated in the 3–4.5-hour window period, 16.4 will have a better outcome and 2.7 will have a worse outcome.^5^ The risk of intracranial haemorrhage was 27 vs 17.6% for placebo.^5^ The mortality between the treated and placebo groups were not significantly different.

The data therefore indicate that the treatment modality is not ideal and is limited by the 3–4.5-hour window period but we have nothing better. IA thrombolysis should theoretically be the better treatment option but strangely, the suggestion in the literature is that IA thrombolysis in not significantly better than IV thrombolysis.^6^ A conclusive study in this regard is lacking.

With regard to mechanical clot retrieval, the simple answer is that this is becoming ‘much ado with nothing significantly attained’. The theoretical concept is appealing and the technical expertise is available. The results, however, and in particular with the popular MERCI device, have been disappointing. The investment in mechanical clot retrieval continues and newer devices are developing, including a penumbra system, which disrupts and aspirates the thrombus. This is undergoing evaluation and is apparently promising. Stent-based therapy and endovascular angioplasty are also used with varying degrees of success.

The problem with these devices is that the endpoint of recanalisation is readily achieved but is not associated with clinically significant outcomes. The future of this therapeutic approach therefore remains to be determined.

The conclusions are that for patients with AIS, early intervention within 4.5 hours with IV r-tPA is the only option for reversal at present. This needs to become widely used in the emergency departments of all public and private hospitals nationally. We must review and recharge the national drive for public stroke awareness to facilitate this.
